# Gender and Sex-Related Differences in Normal Tissue Effects Induced by Platinum Compounds

**DOI:** 10.3390/ph15020255

**Published:** 2022-02-20

**Authors:** Loredana G. Marcu

**Affiliations:** 1 Faculty of Informatics & Science, Department of Physics, University of Oradea, 410087 Oradea, Romania; loredana@marcunet.com; 2 Cancer Research Institute, University of South Australia, Adelaide, SA 5001, Australia

**Keywords:** cisplatin, carboplatin, adverse effects, sex, gender, personalised chemotherapy

## Abstract

Gender medicine in the field of oncology is an under-researched area, despite the existing evidence towards gender-dependent response to therapy and treatment-induced adverse effects. Oncological treatment aims to fulfil its main goal of achieving high tumour control by also protecting normal tissue from acute or chronic damage. Chemotherapy is an important component of cancer treatment, with a large number of drugs being currently in clinical use. Cisplatin is one of the most commonly employed chemotherapeutic agents, used either as a sole drug or in combination with other agents. Cisplatin-induced toxicities are well documented, and they include nephrotoxicity, neurotoxicity, gastrointestinal toxicity, ototoxicity, just to name the most frequent ones. Some of these toxicities have short-term sequelae, while others are irreversible. Furthermore, research showed that there is a strong gender-dependent aspect of side effects caused by the administration of cisplatin. While evidence towards sex differences in animal models is substantial, clinical studies considering sex/gender as a variable factor are limited. This work summarises the current knowledge on sex/gender-related side effects induced by platinum compounds and highlights the gaps in research that require more attention to open new therapeutic possibilities and preventative measures to alleviate normal tissue toxicity and increase patients’ quality of life in both males and females.

## 1. Introduction

As acknowledged by medical research, the important role of sex and gender in influencing treatment outcome is well recognised in several medical fields, however, remains poorly investigated in oncology [1]. This void must be filled with clinical evidence that should originate from interventional studies and trials evaluating sex/gender-specific dosing regimens to counteract the side effects caused by a variety of chemotherapeutic drugs [1].

Platinum-based agents, particularly cisplatin, are one of the most commonly used chemotherapeutic agents, for a large variety of cancers. Cisplatin (cis-diammine-dichloro-platinum) is a heavy metal complex with a central platinum atom surrounded by 2 chloride and 2 ammonia groups. The negatively charged platinum compound becomes positive once inside the cell as the chloride atoms are replaced by water molecules. As the water molecules are easily displaced, the platinum compound undergoes aquation and attaches to the DNA through cross-links, inhibiting cellular function. The powerful cytotoxic mechanism exhibited by cisplatin often comes with a price, as patients frequently become resistant to the drug via various mechanisms: reduced drug accumulation in cells, improved DNA repair, decreased apoptosis and autophagy [2,3]. Furthermore, owing to the heavy metal constituent, platinum agents cause severe normal tissue toxicity, which is often a dose-limiting factor in chemotherapy.

Given that cisplatin is most often administered as part of a combined chemo-radiotherapy regimen, and due to the fact that normal tissue toxicity can be induced by radiation and drug alike, it becomes critical to consider the contribution of each individual therapeutic mechanism to the plethora of adverse effects that were shown to differ among genders [4]. Gastrointestinal toxicity, ototoxicity, nephrotoxicity, neurotoxicity as well as cardiac toxicities were reported among the adverse effects caused by cisplatin and were identified in both males and females, though with varied impacts [4]. Studies investigating sex/gender-dependent tumour response and side effects after radiotherapy are very scarce [5]. Among the biological differences that could explain sex/gender disparity in response to radiotherapy, the following have been identified: recruitment of X-chromosome tumour suppressor genes which escape inactivation, the balance of Th2 (T helper cells) vs. Th1-type cytokines in inflammation, the modulation of an inflammatory response by sex hormones, the protective properties of oestrogen and its receptors, but also the different anatomy and body habitus between man and women [5].

Chemotherapy has a different mechanistic action from radiotherapy, which often results in added toxicity. While the additive or sometimes synergistic effects caused by the radiation-drug interaction are welcome from a tumour control perspective, they become a concern when it comes to healthy organs, especially for long-term or irreversible adverse effects.

The aim of the current work is to collate the evidence towards sex/gender-related differences in normal tissue effects induced by platinum compounds, with a specific focus on the most used platinum compound, cisplatin. Regarding terminology, to account for both the genetic meaning (sex) and the societal context (gender) of the two words, the dual “sex/gender” term will be used when reporting on human studies [6], while for animal studies the term “sex” will be referred to, as gender differences in animal models are much more difficult to detect.

Current evidence towards sex/gender-dependent toxicity after cisplatin chemotherapy is primarily based on pre-clinical research, the number of clinical studies exploring this important aspect being limited. In today’s world of personalised medicine, the investigation of gender-specific effects of cancer therapies should be an essential component of clinical trials, which could be tackled without additional costs or deviations from the initial study design.

## 2. Cisplatin-Induced Normal Tissue Toxicity

The alkylating agent cisplatin is one of the most established and potent chemotherapeutic agents, owing to a number of mechanisms that render this drug efficient in combination with radiation. The most studied modes of cisplatin’s action and interaction with radiation include cellular sensitisation through DNA adduct formation and cell-cycle arrest, inhibition of DNA repair [7], induction of reactive oxygen species triggering cell death, modulation of calcium signalling leading to disruption of cellular function [8] and inhibition of angiogenesis [9].

Cisplatin is successfully administered in a number of cancers, including head and neck, oesophageal, ovarian, lung, testicular and bladder, either as a sole drug or in combination with other chemotherapeutic agents [8]. Cisplatin alone has limited tumoricidal effects, which is the reason why the drug is commonly used in combination with radiotherapy in most of the above-mentioned cancers.

The most frequently reported side effects after cisplatin-based therapy are nephrotoxicity, neurotoxicity, ototoxicity, haematological toxicities, and skeletal muscle dysfunction. While some of them could be overcome to a certain degree with preventative treatment, most side effects are dose-limiting that hinder the overall effectiveness of cisplatin. Ototoxicity is a common side effect without known preventative measures that affects nearly all patients treated with cisplatin. Hearing loss is often bilateral and permanent, with audiometric studies reporting an elevation of hearing threshold in 75–100% of patients after cisplatin chemotherapy [10]. Ototoxicity can worsen in patients that, besides cisplatin, receive radiotherapy at the head and neck region [11]. More recent studies investigating pharmacogenetic factors as predictors of cisplatin-induced ototoxicity in head and neck cancer patients revealed that COMT (catechol-O-methyltransferase) allele carriers had higher ototoxicity risk, whereas carriers of MATE1 (multidrug and toxin extrusion) allele were better protected from such side effects [12]. Research shows that COMT and MATE1 genotyping could lead to strategies for mitigating ototoxicity caused by cisplatin.

Neurotoxicity induced by cisplatin manifests as a sensory peripheral neuropathy characterised by distal axon degeneration in hands and feet and is a major dose-limiting effect that persists over the years, often with incomplete recovery. Studies showed a correlation between the severity of neuropathy and the cumulative dose of cisplatin [13]. The latest research has identified the mechanisms behind this side effect showing that neurotoxicity is mediated by SARM1 (sterile alpha and toll/interleukin-1 receptor motif-containing 1), a key regulator of Wallerian degeneration (i.e., retrograde degeneration of the distal end of axons) and activation of calpains that mediate the breakdown of the axonal cytoskeleton [14]. As with ototoxicity, there are no efficient measures to prevent neurotoxicity or to treat neurosensory damage induced by cisplatin. This is an added reason for a more in-depth evaluation of sex/gender-related toxicities to find specific ways to ameliorate or possibly avoid these side effects.

Cisplatin-induced nephrotoxicity is a dose-dependent side effect observed in up to 40% of patients, manifested as acute kidney injury due to tubular dysfunction [15]. It is caused by the accumulation of the heavy metal platinum in renal epithelial cells, through the formation of platinum-DNA adducts that are toxic to dividing cells. Studies on gamma-glutamyl transpeptidase showed that this enzyme plays an important role in increasing resistance to cisplatin in tumour cells, whereas in renal cells the expression of the enzyme rendered the cells sensitive to the drug [16]. A number of studies confirmed the involvement of the p53 protein in cisplatin-caused nephrotoxicity, interfering with molecules that have renoprotective functions. While several strategies have been proposed to protect kidney functions against the toxic effects of cisplatin, it was proven difficult to identify molecular candidates that do not limit cisplatin’s tumoricidal effects in favour of renal protection [17].

As with most conventional chemotherapeutic agents that affect rapidly proliferating cells, gastrointestinal toxicities are also common after cisplatin administration. Cisplatin causes emesis 24 h-post-therapy in most patients. Anorexia, diarrhoea, and malabsorption are common gastrointestinal effects that can result in weight loss. While reversible, gastrointestinal toxicity is often a cause for treatment interruption, affecting the overall therapeutic effect of cisplatin. Efficient gastroprotective measures are still lacking [18].

Weight loss owing to muscle wasting is another side effect observed in patients treated with cisplatin. Skeletal muscle dysfunction, particularly muscle mass depletion, is an adverse effect caused by cisplatin that deserves more consideration in order to preserve muscle mass and increase patients’ quality of life during therapy. Furthermore, muscle wasting was shown to be a negative predictor of treatment outcome, and is correlated with increased mortality [19]. Muscle dysfunction induced by cisplatin has several underlying mechanisms, including lipid metabolism dysregulation, reduction in protein synthesis and activation of proteolysis, mitochondrial damage, the upregulation of pro-inflammatory cytokines, oxidative stress, and calcium homeostasis [19].

## 3. Sex/Gender-Difference in Normal Tissue Toxicity Induced by Cisplatin

Most toxicity studies to evaluate sex/gender-dependent variations were undertaken on animal models. In humans, the vast majority of reports are retrospective analyses of clinical studies/trials, without an initial consideration of sex/gender as a variable that requires distinctive assessment.

### 3.1. Animal Studies

Cisplatin is known to cause acute kidney injury due to its cumulative and dose-dependent effect, leading to the activation of multiple pro-inflammatory cytokines and infiltration of inflammatory cells into the kidney. Furthermore, endothelial dysfunction is another cisplatin-induced renal effect that causes vasoconstriction and, in turn, tubular cell injury. Nitric oxide is known to play a role in renal hemodynamics, and thus in the evolution of kidney disease, which was shown to vary between genders [20]. While the underlying mechanisms are not fully elucidated, it is thought that the reduced availability of nitric oxide in males, over time, contributes to a decrease in renal plasma flow and an acceleration of pre-existent kidney disease [21]. Furthermore, studies in animal models showed that renal vasculature in males may be more dependent on nitric oxide than that of females. Animal models were therefore used to investigate the mechanisms behind sex-related nephrotoxicity induced by cisplatin and also to explore protective measures against kidney toxicity [22,23,24,25,26,27,28,29].

In the experiment reported by Nematbakhsh et al., Wistar rats of both sexes were used to evaluate the level of nephrotoxicity after two weeks of treatment with cisplatin (1 mg/kg/day) [24]. Except for the serum levels of nitric oxide and malondialdehyde, all other biochemical markers related to kidney function were significantly elevated in males (*p* < 0.05), and results, confirmed by pathological evaluations, revealed greater nephrotoxic intensity in male rats. A different drug schedule was tested by Pezeshki et al., with a single large dose of cisplatin administered to Wistar rats (7.5 mg/kg) [28]. The study focused not only on sex differences but on age-related side effects as well. The results showed significant variations between old (16–20 weeks old) and young male rats (10 weeks old) with greater toxicities in old males than in female rats, while young males exhibited the best toxicity profile. The study confirms the importance of considering age, next to sex, when analysing toxicity data.

A comparative drug scheduling protocol with single (7.5 mg/kg) versus daily cisplatin (3 mg/kg/day) for 5 days showed sex-dependent alterations of creatinine and blood urea nitrogen levels for both protocols, with significantly higher levels in female rats (*p* < 0.05) but also significant differences between the two protocols [29]. Differences in natrium excretion between protocols were also observed as well as differences in kidney weight and kidney tissue damage scores, with an important alteration in the group treated with a continuous dose of cisplatin (*p* < 0.05). The treatment protocol is proven to be another influencing factor of renal function and toxicity induced by cisplatin.

The mechanisms behind sex-dependent nephrotoxicity are not fully elucidated, though differences in renal circulation between males and females could be a plausible factor [24]. Another hypothesis is linked to drug uptake by the kidneys, showing higher concentrations than in blood, which is suggestive of an active accumulation of cisplatin by the kidneys. The passage of cisplatin into cells occurs via two membrane transporters—the copper transporter (Ctr1) and the organic cation transporter (OCT2). OCT2 levels were reported to be significantly higher in males and were positively correlated with nephrotoxicity [26].

A number of studies investigated diverse agents for their potential nephroprotective effects when co-administered with cisplatin. To counteract the endothelial toxicity caused by cisplatin, L-arginine was used in Wistar rats to promote endothelial cell function [22]. The study aimed to assess kidney health through blood urea nitrogen, creatinine, and nitrite level measurements, using gender as a variable. According to the results, L-arginine did not decrease the levels of blood urea nitrogen and creatinine induced by cisplatin in female rats. This is thought to be due to the role of oestrogen in inducing nitric oxide production and elevating the activity of the nitric oxide synthase enzyme, which might interfere with the effect of L-arginine. Nevertheless, in male rats, the protective role of L-arginine was proven, showing lower toxicity levels in the treated rats than those receiving cisplatin only. Further to the previous study, the same research group evaluated the protective role of angiotensin II receptor 1 blockade (losartan) against cisplatin-caused nephrotoxicity, obtaining similar results in terms of sex-based protection [23]. Female rats treated with cisplatin and losartan showed significantly greater serum levels of blood urea nitrogen and creatinine (*p* < 0.05), suggesting that losartan promotes cisplatin-induced kidney damage related to renin-angiotensin system receptors which are known to have a sex-dependent action. Contrastingly, in male rats, losartan exhibited protective effects against renal toxicity.

Another agent with potential nephroprotective effects tested in rats was bosentan, a nonselective endothelin-1 receptor antagonist with known vasodilatory effects, that was assumed to reduce the high levels of endothelin-1 induced by cisplatin, a leading cause of vasoconstriction in the kidneys. Jokar et al. have tested bosentan on rats of both sexes, the study showing no nephroprotective effects, irrespective of sex [25]. Overall, female rats exhibited a higher degree of kidney injury than males, an observation that is in agreement with previously reported results. Similar findings were reported with enalapril, an angiotensin-converting enzyme inhibitor that was administered to both male and female Wistar rats as a renoprotective agent [27]. Besides failing to alleviate cisplatin-related side effects, enalapril significantly increased nephrotoxicity in female rats (*p* < 0.05), probably owing to the sex-dependent function of the renin-angiotensin system.

Ototoxicity in animal models was investigated in a couple of studies. Kirkim et al. conducted their investigation in 14 male and 14 female Wistar albino rats, each with a control and a treated subgroup, to evaluate auditory brainstem response and distortion product otoacoustic emission to reflect the cochlear function [30]. The hearing of female rats more significantly deteriorated, as indicated by a pronounced spiral ganglion and brainstem tissue damage.

To evaluate neuropathic effects, cold, and mechanical allodynia were studied in both male and female mice after cisplatin treatment for seven consecutive days, revealing no sex differences in the manifestation of these side effects [31]. Contrastingly, Wongtawatchai et al. found that sex differences in diverse aspects of cisplatin neurotoxicity exist, with certain side effects being more pronounced in males while other toxicities were more striking in females [32] (see Table 1).

Clinical settings are nevertheless required to elucidate the underlying mechanisms for the differential response of normal tissue to cisplatin between the two sexes.

### 3.2. Patient Studies

To date, animal studies on sex-dependent toxicity outweigh clinical studies, which are very rarely designed to specifically target gender medicine. A report of the Eastern Cooperative Oncology Group trial looking at survival differences by sex/gender in patients with advanced non-small-cell lung cancer (NSCLC) treated with cisplatin-based chemotherapy, also analysed sex/gender-dependent adverse effects [33]. Overall, females presented with more severe toxicity than males, with higher rates of gastrointestinal and neurologic effects. Neurosensory deficits were reported in 10% females vs. 7% males (*p* = 0.06), with more significant differences in gastric effects: nausea (33% vs. 20%, *p* < 0.0001) and vomiting (30% vs. 18%, *p* < 0.0001). The only side effect that was more prominent in men was cardiac toxicity (4.1% females vs. 7.6% males, *p* = 0.02).

A retrospective analysis based on Taiwan’s National Health Insurance Research Database (3973 men and 1154 women) aiming to evaluate the association between sex hormones and cisplatin-induced nephrotoxicity showed a higher percentage of women (39.08%) than men (36.95%) diagnosed with kidney disease after treatment with cisplatin [4]. The highest risk was observed among perimenopausal women, with a hazard ratio of 1.28 (95% CI: 1.02–1.61) as compared to men, correlated with higher levels of estradiol shown in this group. The study highlights the protective role of oestrogen in younger females and recommends additional studies to validate the findings and to elucidate the influence of sex hormones on nephrotoxicity differences among genders and also among females from various age groups.

Paediatric patients treated with cisplatin are of particular interest regarding ototoxicity. Studies showed that platinum-based chemotherapy can lead to long-term sequelae that influence the social development and academic performance of the affected children, males being more predisposed to ototoxicity than females [34,35]. A retrospective evaluation of hearing loss among 102 paediatric patients treated with cisplatin for various malignancies showed a 42% hearing loss and 28% moderate to severe ototoxicity, with males exhibiting a significantly higher risk than females (*p* = 0.005, OR 4.812) [35]. Additionally, age and cumulative doses of cisplatin were identified as other risk factors for ototoxicity, with very young patients (mean age of 4.5 years) presenting higher-grade side effects than older children. It is considered that sex hormones, particularly oestrogen and its signalling pathways, are responsible for the protective effect against hearing loss in female patients, as the level of oestrogen positively affects otoacoustic emission amplitudes and also the auditory brainstem response wave latencies [36].

These sex/gender-based differences in side effects are most likely due to variations in expression levels of metabolic enzymes in the kidney and liver, thus in pharmacokinetic differences of cisplatin in males and females [37]. Studies showed a positive correlation between cisplatin metabolism in different organs and specific glutathione S-transferases activities, suggesting that this enzyme strongly influences both the uptake and retention of the drug by kidneys and liver [38]. In females, the higher cisplatin-induced toxicity is caused by the higher glutathione S-transferases activity that leads to a longer biological half-life and higher uptake and retention of the drug in targeted organs [39].

**Figure 1:**
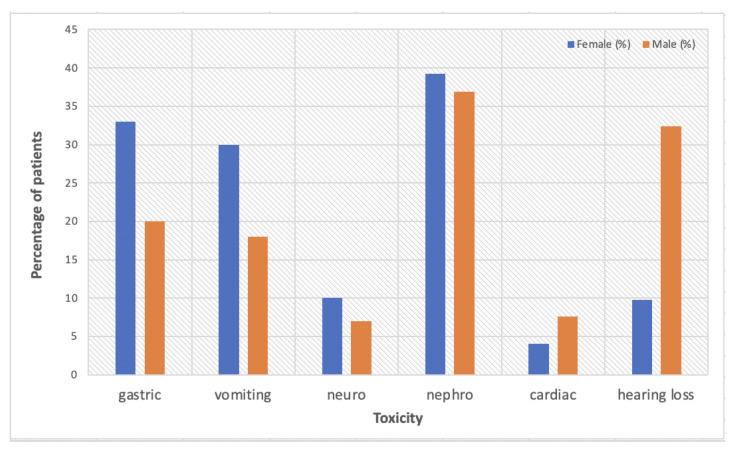
A summary of sex/gender-dependent cisplatin-based toxicity data reported by the current literature [4,33,35].

Figure 1 is a summary of sex/gender-based toxicity data reported by the current literature.

## 4. Platinum-Based Alternative Compounds to Reduce Normal Tissue Toxicity

To overcome some of the normal tissue toxicity induced by cisplatin but to retain the platinum-caused tumour effects, several other platinum-based compounds have been developed over the years, yet none as popular as cisplatin. Carboplatin, the second generation of platinum-based drugs was shown to be better tolerated, due to its different toxicity profile compared to cisplatin. Due to lower protein binding of carboplatin, as compared to cisplatin, the former has a longer half-life of ultrafilterable platinum as well as higher cumulative urinary platinum excretion, leading to better renal tolerance and lower rates of nephrotoxicity among patients treated with carboplatin [40]. The high risk of gastric effects, ototoxicity, and nephrotoxicity induced by cisplatin is replaced by myelosuppression and neurotoxicity with the administration of carboplatin, though the latter is often similar to cisplatin-caused neuropathy [41]. However, the inferior effectiveness on tumour response compared to cisplatin lead to limited employment of the new agent in a number of cancers [42,43].

Owing to its reduced nephrotoxic and neurotoxic effects in pre-clinical studies and for its potential efficacy against a number of cisplatin-resistant cells lines, tetraplatin (also known as ormaplatin) was trialled in the 90s [44]. Nevertheless, the drug failed in its attempt to attain clinical usage when several first phase trials showed contrasting results to pre-clinical studies [44,45]. Lobaplatin is a diastereomeric mixture of platinum (II) complexes that underwent phase II testing, showing cytotoxic potential in several tumour types. In pre-clinical models, lobaplatin seemed to overcome resistance to cisplatin and carboplatin [46]. It is, however, an under-investigated drug, with several unexplored applications, including its combination with radiotherapy or other chemotherapeutic agents.

The third generation of the platinum drug family is represented by oxaliplatin, which exhibits similar radiosensitising properties to cisplatin, though with somewhat reduced normal tissue toxicity [47]. While kidney toxicity is not a dose-limiting factor in patients treated with oxaliplatin, owing to adequate glomerular filtration and clearance, neurological dysfunctions are often a cause for treatment cessation. Oxaliplatin was found to add unique neurotoxic effects to the list of adverse effects caused by platinum compounds, in the form of a rapid onset of painful peripheral neuropathy that is aggravated when the patient is exposed to cold [48]. Additionally, there were conflicting results on the efficacy of oxaliplatin on cisplatin-resistant cell lines; the reason why the use of this drug in particular cancers require more convincing evidence [47]. In order to decrease nephrotoxicity and gastrointestinal side effects caused by cisplatin, another analogue drug was developed—nedaplatin—with equivalent efficacy in terms of response rate. The drug has gained more popularity in certain Asian countries, as reported by successful clinical trials, particularly on nasopharyngeal carcinoma [49,50,51].

Mitaplatin was developed as a fusion between cisplatin and the orphan drug dichloroacetate and was shown to display toxic effects on cisplatin-resistant cancer cells [52]. Furthermore, mitaplatin demonstrated better tumour cell selectivity compared to cisplatin by inducing apoptosis without affecting healthy cells, thus potentially reducing normal tissue side effects [53]. Furthermore, a mitochondrial-based difference between cisplatin-sensitive and cisplatin-resistant cells allowed mitaplatin to increase the sensitivity of resistant cells by provoking mitochondrial impairment [53]. However, reports on the use and effectiveness of mitaplatin are scarce, with further research required to prove its clinical efficacy.

The fourth generation of platinum agents includes the first orally administered compounds (as compared to the intravenous route for all the above agents), such as satraplatin. The major advantages of satraplatin consist of a better toxicity profile and a potent activity against cisplatin-resistant tumours [54]. While it underwent phase III trial in pretreated metastatic castrate-resistant prostate cancer, it needs testing in other cancer types where cisplatin showed efficiency. Table 2 is a compilation of platinum compounds that have been developed to date.

## 5. Sex/Gender-Difference in Normal Tissue Toxicity Induced by Other Platinum Compounds

### 5.1. Animal Studies

Studies investigating sex-dependent differences in normal tissue toxicity after treatment with platinum compounds other than cisplatin are scarce. A recent pre-clinical investigation using rodent models aimed to determine the impact of dose, sex, and strain on oxaliplatin-caused peripheral neuropathy [56]. The study was prompted by the lack of efficient therapies to overcome long-term platinum-induced peripheral neuropathy, one of the most common toxicities reported in the clinics. The investigation consisted of a complex evaluation of behavioural, sensory, morphometric and electrophysiologic processes in both sexes of the two inbred mouse strains accrued by the study (C57BL/6J and BALB/cJ). While differences between strains were identified for various endpoints, sex-specific effects were also identified. C57BL/6J female mice presented anxiety-like behaviour after treatment completion and developed mechanical hypersensitivity, whereas BALB/cJ females showed a significant reduction in nerve conduction amplitude one week post-therapy and considerable reduction in intraepidermal nerve fibre density compared to their male counterparts. High-dose oxaliplatin resulted in a more significant body weight loss in C57BL/6J male than female mice (*p* = 0.014). The study suggests the important role of sex, strain, and assay type when investigating sex-dependent effects in animal models to develop potential therapeutic approaches.

### 5.2. Patient Studies

Patient studies on sex/gender-dependent response to platinum compounds other than cisplatin are also limited, and in most reported cases the platinum agent is given in combination with other chemotherapeutic drugs, which can induce bias in data interpretation and influence the outcome. A few Japanese studies aimed to determine whether sex/gender is a differentiating factor between tumour response and normal tissue toxicity among patients treated for non-small-cell lung cancer (NSCLC). In a retrospective clinical study reported by Yamamoto et al., a combination between carboplatin and paclitaxel was administered to 227 unresectable stage IIIB-IV NSCLC patients (147 males and 80 females), showing that in both sexes/genders the response rate reached 39% [57]. Female sex was correlated with a more favourable prognosis (median progression-free survival of 5.3 months in females vs. 4.4 months in males), however, females exhibited more severe haematological toxicities than their male counterparts. Similar results regarding toxicity were reported by another Japanese study investigating toxicity after combined platinum-gemcitabine chemotherapy for NSCLC [58]. This small retrospective study involved 34 patients (22 males and 12 females), showing significantly higher rates of leucopenia (*p* = 0.013) and neutropenia (*p* = 0.039) among female patients, suggesting the need for adjustment in dosage based on sex/gender.

Females were also more prone to develop pulmonary toxicities after carboplatin administration [59]. A prospective study on lung toxicity in patients treated with carboplatin/gemcitabine that involved assessment of pulmonary symptoms, pulmonary function tests, arterial blood gases, and radiography-based lung evaluation revealed significant changes in post-therapy diffusion capacity for carbon monoxide in females, which, nevertheless, was shown to be reversible. Based on a survey conducted in 514 patients that underwent standard dose chemotherapy over 2 weeks, carboplatin was also correlated with more severe oral mucositis in females (*p* = 0.03) [60].

A recent study investigated allergic reactions as a side effect of platinum agents among genders, a less commonly assessed effect, which was shown to be more frequent in females [61]. Of 1090 patients treated with platinum-based agents enrolled in the study, 35 patients were found to be allergic to platinum compounds, with the most pronounced reactions observed in females treated with carboplatin (*p* = 0.034). Blood cell count revealed a statistically significant increase in neutrophils (*p* = 0.06) and a decrease in monocytes (*p* = 0.023) in female patients after carboplatin administration, being indicative of allergic reactions. Treatment with oxaliplatin also triggered allergic reactions that were more frequent and pronounced in females than in male patients [62].

## 6. Discussion and Future Prospects

Gender medicine is not a novel concept, yet it has come to the attention of the research community more recently, which explains the scarce evidence in certain areas of oncology, such as chemo-radiotherapy. While disparities in drug response between sexes and genders are documented [4,33,34,35,36], sex/gender is very rarely the main focus of a study, most often being just a variable added at a later stage for data analysis. To advance the field of gender medicine and to avoid unnecessary side effects and unwanted outcomes after chemotherapy, sex/gender should be considered in pre-clinical as well as cohort studies, and also included as a matched variable in clinical trials.

The number of investigations evaluating sex/gender-dependent toxicity in humans after platinum-based chemotherapy is scarce. While useful on several levels, animal studies have their limitations in translating the findings to humans, as they cannot mimic the complex processes that undergo in the human body. Moreover, toxicity outcomes, as revealed by mice studies, are strain-dependent, which further complicates the interpretation of results.

Since it is documented that cisplatin-induced side effects are cumulative and dose-dependent, it would be useful to have more studies investigating daily low-dose versus weekly high-dose administration of cisplatin in animal models to assess the normal tissue effects in the sex/gender context. While some clinical studies have succeeded in showing a better toxicity profile with daily low doses of cisplatin as compared to weekly administration, none investigated the impact of drug scheduling on sex/gender-related toxicities [63].

Another limitation of the above-described clinical studies consists of the combined administration of drugs, hindering the unbiased interpretation of single-drug-induced toxicity. However, despite all limitations, the results of both animal and human studies support the conclusion whereby normal tissue toxicity after platinum drugs is sex/gender-dependent, and requires further investigations within well designed clinical trials.

Until additional information becomes available on gender oncology, there are accessible measures to mitigate adverse effects, which are applicable as part of a routine chemotherapy protocol, irrespective of sex/gender. One such measure is chronotherapy, the synchronised drug delivery with the patient’s circadian rhythm. The evidence-based fact that certain chemotherapeutic agents induce fewer side effects when administered at night while others are more efficient on tumour cells when given daytime, should be sufficiently convincing to take the necessary steps to regard chronotherapy as the fourth dimension of oncological treatment [64]. For instance, a recent study on the efficacy and safety of cisplatin chronotherapy in rats with ovarian carcinoma reported less toxicity with the morning administration of the drug as compared to evening chemotherapy, including nephrotoxicity, liver toxicity and gastrointestinal toxicity [65]. This observation confirms the results of other chronotherapy studies on the platinum toxicity profile, where morning cisplatin led to less severe peripheral neuropathy as compared to evening treatment [66].

To mitigate drug-induced side effects, the scientific community has provided evidence for the role of pharmacogenomics in personalised chemotherapy. Pharmacogenomics is a relatively new research field that aims to explore the genetic basis of an individualised response to drugs and to offer cancer patients a more customised chemotherapeutic regimen, compatible with their genetic makeup. A large number of gene mutations have been identified in various cancers to be responsible for drug resistance and toxicity. Within a preliminary study on genetic variability and drug-induced toxicity after platinum-based chemotherapy, a significant association between the variants of glutathione S-transferase Mu 1 (GSTM1) and cisplatin toxicity were observed (*p* = 0.043) [67]. Nephrotoxicity induced by cisplatin was also shown to have a genetic component. Through a systematic review of the literature, over 300 single nucleotide polymorphisms (SNPs) across 135 genes were studied, of which 29 SNPs in 14 genes showed a significant correlation with cisplatin-induced nephrotoxicity [68]. Variants of two genes involved in DNA repair, ERCC1 and ERCC2 (the excision repair cross-complementation group), were consistently correlated with an increased risk of nephrotoxicity, while polymorphisms in the SLC22A2 (solute carrier family 22 member 2) gene that mediates platinum uptake by the kidney were identified as protective factors against renal toxicity and were the most promising candidate genes in predicting nephrotoxicity after cisplatin therapy.

Although pharmacogenomic research is advancing at a fast pace, the implementation of results is overdue. In order to support clinical implementation of pharmacogenomics, international large-scale enterprises, such as the Clinical Pharmacogenetics Implementation Consortium (CPIC) (USA) and the Ubiquitous Pharmacogenomics program (U-PGx) (Europe) facilitate the bench to bedside translation of drug-gene interaction research [69].

To sum up, the following steps could lead towards a more personalised approach in oncology, and particularly in cisplatin-based chemotherapy, to manage variations in normal tissue response that can differently affect males and females but also to reduce side effects in both genders:

1. Consider sex/gender as a matched variable in clinical trials;
2. Identify differences among sexes/genders in both tumour response and normal tissue toxicity;
3. Implement solutions to alleviate the impact of cisplatin-based chemotherapy on normal tissues:
  (a) Find alternative platinum agents with similar cytotoxic effects on the tumour.
  (b) Use chronotherapy principles when administering platinum compounds.
  (c) Consider dose de-escalation without compromising tumour response.
  (d) Use pharmacogenomics for personalised chemotherapy.

## Figures and Tables

**Table 1:** Compilation of studies investigating sex-dependent normal tissue toxicity induced by cisplatin in animal models.

Investigated Toxicity	Investigated Parameters	Gender-Dependent Effects	Study [Ref.]
Nephrotoxicity in Wistar rats	Serum creatinine, blood urea nitrogen, nitric oxide metabolite, malondialdehyde	Male rats: significantly greater levels of serum creatinine, blood urea nitrogen, malondialdehyde; also, greater kidney damage score (*p* < 0.05).	Nematbakhsh et al., 2013 [24]
Nephrotoxicity in Wistar rats	Serum creatinine, blood urea nitrogen, kidney weight, kidney tissue damage score	Male rats: greater increase in blood urea nitrogen. Both sexes: significant body weight loss, increased serum levels of creatinine, blood urea nitrogen.	Zamani et al., 2016 [27]
Nephrotoxicity in Wistar rats	Serum creatinine, blood urea nitrogen, aspartate aminotransferase, alkaline phosphatase, nitrite, kidney weight malondialdehyde	Male rats (young): lower blood urea nitrogen and creatinine than females; the highest creatinine clearance. Male rats (old): greater levels of serum creatinine, blood urea nitrogen, and kidney damage score than females.	Pezeshki et al., 2017 [28]
Nephrotoxicity in Wistar rats	Creatinine levels, blood urea nitrogen levels, sodium excretion	Female rats: significantly greater levels of serum creatinine and blood urea nitrogen.	Jilanchi et al., 2018 [29]
Ototoxicity in Wistar albino rats	Distortion product otoacoustic emission; Auditory brainstem response	Female rats: more pronounced hearing toxicity.	Kirkim et al., 2015 [30]
Neuropathic pain in mice	Cold/mechanical allodynia	No sex-related differences in cold or mechanical allodynia were observed.	Naji-Esfahani et al., 2016 [31]
Neuropathy in rats	Heat latency of hind paw; sciatic motor nerve conduction velocity; Pathological alterations in the sciatic nerve and dorsal root ganglion	Male rats: higher severity of weight loss, prolonged heat latency, slow motor nerve conduction velocity, atrophy of neuronal cell body and nucleus. Female rats: more significant reduction in myelinated fiber diameter and density, myelin thickness.	Wongtawatchai et al., 2009 [32]

**Table 2:** Platinum compounds as chemotherapeutic agents with their possible advantages and therapeutic limitations as compared to cisplatin (adapted from [55]).

Platinum Compound	Benefits	Limitations	Normal Tissue Toxicity
Cisplatin	Potent cytotoxicity Most trialled platinum agent	High normal tissue toxicity Drug resistance	Nephrotoxicity Ototoxicity Neurotoxicity Gastrointestinal
Carboplatin	Reduced normal tissue toxicity (no nephrotoxicity).	Inferior tumour response rate.	Myelosuppression
Oxaliplatin	Reduced normal tissue toxicity and better tolerability. Greater cytotoxicity and inhibition of DNA synthesis.	Conflicting results on the efficacy on cisplatin-resistant cell lines.	Neurotoxicity Hematologic Gastrointestinal
Nedaplatin	Reduced nephrotoxicity and gastrointestinal toxicity. Similar tumour control.	Often exhibits cross-resistance with cisplatin thus its clinical application is limited.	Thrombocytopenia
Mitaplatin	Exhibits toxic effects on cisplatin-resistant head and neck tumour cells. Better selectivity for tumour cells than cisplatin.	More research is needed to prove its clinical efficacy.	Neurotoxicity Hepatotoxicity (possible toxicities, not studied in humans)
Enloplatin	Tested in the 90 s without successful clinical implementation		
Lobaplatin	Shows activity in various tumour types. Overcomes certain forms of cisplatin/carboplatin resistance.	Underexplored agent, needs trialling in combination with radiation.	Thrombocytopenia
Satraplatin	Efficient in cisplatin-resistant cell lines. Better toxicity profile than cisplatin.	New generation of orally active platinum agents. More investigations are needed.	Carboplatin-like toxicity profile
Tetraplatin/ormaplatin	Tested in the 90 s; under investigation by some research groups		

## Data Availability

Data sharing not applicable.
